# miR-2110 orchestrates ERK–ELK1 transcriptional repression to induce cell-cycle arrest and enhance cytarabine sensitivity in acute myeloid leukemia

**DOI:** 10.3389/fonc.2026.1833649

**Published:** 2026-05-25

**Authors:** Ya Li, Ying Chen, Aoshuang Huang, Yun Zhan, Ming Ni, Fengqi Zhang, Shaofei Xu, Yanju Li

**Affiliations:** Department of Hematology, Affiliated Hospital of Guizhou Medical University, Guiyang, Guizhou, China

**Keywords:** acute myeloid leukemia, chemoresistance, cytarabine sensitivity, ELK1, microRNA, miR-2110

## Abstract

**Introduction:**

Acute myeloid leukemia (AML) remains a heterogeneous hematologic malignancy with frequent therapeutic resistance and relapse. Although miR-2110 is upregulated in AML, its functional role and downstream mechanism remain unclear.

**Methods:**

Publicly available miRNA-sequencing data were analyzed to identify dysregulated miRNAs in AML. miR-2110 expression was validated in AML cell lines. Functional assays, including CCK-8, EdU incorporation, apoptosis analysis, western blotting, dual-luciferase reporter assays, rescue experiments, Ara-C sensitivity assays, and xenograft experiments, were performed to evaluate the role of miR-2110 and its downstream target.

**Results:**

miR-2110 was upregulated in AML samples and cell lines but exerted tumor-suppressive effects. miR-2110 overexpression inhibited AML cell proliferation, reduced cell-cycle–related protein expression, and promoted apoptosis-related changes. ELK1 was identified and validated as a direct target of miR-2110 through multiple predicted binding sites within its 3′UTR. Restoration of ELK1 partially reversed miR-2110-associated molecular changes, supporting ELK1 as an important downstream mediator. miR-2110 also enhanced Ara-C responsiveness in parental and resistant AML cells, while Ara-C-resistant cells showed reduced miR-2110 and increased ELK1 expression. *In vivo*, miR-2110 overexpression suppressed xenograft tumor growth without obvious body weight loss.

**Discussion:**

miR-2110 functions as an upregulated but tumor-suppressive miRNA in AML. The miR-2110–ELK1 axis contributes to regulation of AML cell growth, apoptosis-related signaling, and Ara-C responsiveness, providing a potential molecular basis for further investigation of miRNA-mediated therapeutic modulation in AML.

## Introduction

Acute myeloid leukemia (AML) is a highly heterogeneous hematologic malignancy characterized by uncontrolled proliferation of immature myeloid cells and profound disruption of normal hematopoiesis (1, 2). Despite advances in risk stratification and treatment strategies, therapeutic resistance and disease relapse remain major clinical challenges, particularly in pediatric AML, where biological features differ substantially from those observed in adult disease (3–5). A deeper understanding of the molecular programs governing leukemic cell survival, transcriptional dependency, and drug response is therefore essential for improving therapeutic outcomes.

MicroRNAs (miRNAs) are increasingly recognized as important regulators of gene expression networks in leukemia; however, the functional interpretation of miRNA dysregulation remains complex (6–8). Notably, altered miRNA expression does not necessarily reflect oncogenic activity. In certain pathological contexts, miRNAs may be transcriptionally induced as part of a compensatory or stress-responsive program, while functionally exerting tumor-suppressive effects by restraining aberrant signaling pathways (9). In leukemia, several signaling pathways have been implicated in mediating these context-dependent miRNA effects. For example, the MAPK/ERK pathway plays a central role in regulating proliferation and survival, while PI3K/AKT signaling contributes to metabolic adaptation and anti-apoptotic responses (10, 11). In parallel, transcriptional regulatory networks involving immediate early genes and stress-responsive pathways further integrate extracellular stimuli into leukemic cell fate decisions (12). Dysregulation of these interconnected pathways is frequently observed in AML and provides a functional framework through which miRNAs may exert either oncogenic or tumor-suppressive roles depending on cellular context. Failure of such compensatory mechanisms may contribute to disease progression despite elevated miRNA levels. This context-dependent behavior underscores the necessity of functional and mechanistic validation beyond expression profiling alone.

Transcription factors downstream of mitogen-activated protein kinase (MAPK) signaling represent critical nodes in leukemic biology, integrating extracellular cues into gene expression programs that promote cell-cycle progression and survival (13). ELK1, a member of the ETS transcription factor family, is a well-characterized MAPK-responsive factor that regulates immediate early genes and proliferation-associated transcription (14). While aberrant MAPK–ELK1 activity has been implicated in malignant transformation, the post-transcriptional regulatory mechanisms controlling ELK1 expression in AML remain incompletely understood (15, 16).

In the present study, we aimed to identify miRNAs that, despite being upregulated in leukemia, functionally act to constrain oncogenic transcriptional programs and influence therapeutic response in AML. Ara-C remains a cornerstone chemotherapeutic agent in AML treatment. As a deoxycytidine analog, Ara-C is incorporated into DNA during the S phase of the cell cycle, leading to chain termination and inhibition of DNA synthesis, ultimately triggering DNA damage–associated apoptosis. Despite its widespread clinical use, intrinsic and acquired resistance to Ara-C frequently limits therapeutic efficacy (17, 18). Understanding the molecular determinants that modulate Ara-C sensitivity, particularly those linked to transcriptional regulation and cell-cycle control, is therefore of considerable clinical importance. By analyzing the miRNA-sequencing dataset GSE196886, which compares pediatric AML samples with umbilical cord blood controls, we identified miR-2110 as a consistently upregulated miRNA in leukemia. Through an integrative strategy combining multi-database target prediction, direct molecular validation, network-based analyzes, and functional assays, we demonstrate that miR-2110 directly targets ELK1 and attenuates MAPK-associated transcriptional activity. Importantly, enforced expression of miR-2110 suppressed leukemic cell proliferation, promoted apoptosis, and enhanced sensitivity to Ara-C, indicating a tumor-suppressive role despite its elevated expression in AML. A simplified *in vivo* model further supported these findings. Collectively, this study defines a miR-2110–ELK1 regulatory axis that links compensatory miRNA upregulation to transcriptional restraint and chemosensitivity in AML, providing mechanistic insight beyond conventional miRNA expression studies.

## Materials and methods

### Public miRNA sequencing data and differential expression analysis

Publicly available miRNA expression data were obtained from the Gene Expression Omnibus (GEO) database under accession number GSE196886, which comprises pediatric acute myeloid leukemia samples and umbilical cord blood controls. This dataset was generated using high-throughput small RNA sequencing (miRNA-seq), enabling comprehensive profiling of mature miRNA expression. Raw count matrices were downloaded and processed using R software. Differential expression analysis was performed using the limma package after appropriate normalization, with thresholds set at |log_2_ fold change| > 1 and adjusted P < 0.05. Volcano plots were generated to visualize expression patterns, and candidate miRNAs were prioritized for further validation. The GSE196886 dataset was selected for this study due to its focus on pediatric AML samples, availability of genome-wide miRNA expression profiles, and inclusion of matched control samples, which together provide a suitable framework for identifying differentially expressed miRNAs relevant to disease context.

### Target prediction and bioinformatic analysis

Putative downstream targets of miR-2110 were predicted using multiple independent databases, including TargetScan, miRWalk, miRDB, and DIANA-microT-CDS. Only genes predicted by at least three algorithms were retained for further analysis. Gene Ontology (GO) and Kyoto Encyclopedia of Genes and Genomes (KEGG) enrichment analyzes were performed using the clusterProfiler R package. Protein–protein interaction (PPI) networks were constructed based on the STRING database and visualized using Cytoscape software. Core interaction modules were extracted using the MCODE plugin.

### Cell culture

Human AML cell lines THP-1, HL-60, as well as the human bone marrow stromal cell line HS-5, were obtained from commercial cell repositories. THP-1 and HL-60 cells were maintained in RPMI-1640 medium supplemented with 10% fetal bovine serum (FBS), while HS-5 cells were cultured in DMEM containing 10% FBS. All cells were incubated at 37 °C in a humidified atmosphere containing 5% CO_2_ and routinely tested to confirm the absence of mycoplasma contamination.

### Cell transfection

miR-2110 mimics, negative control (NC) oligonucleotides, and ELK1 overexpression plasmids, along with their corresponding control vectors, were purchased from Sangon Biotech (Shanghai, China). The miR-2110 mimic sequence was “ 5′-UUGGGGAAACGGCCGCUGAGUG-3′”. The negative control oligonucleotides were designed as non-targeting sequences with no homology to human genes. For ELK1 overexpression, the full-length human ELK1 coding sequence was cloned into a pcDNA3.1(+) expression vector (Invitrogen, USA), and the empty pcDNA3.1 vector was used as a control. Transfections were performed using Lipofectamine 3000 (Thermo Fisher Scientific, USA)according to the manufacturer’s instructions. Cells were harvested 24 h after transfection for subsequent assays.

### Quantitative real-time PCR

Total RNA was extracted using TRIzol reagent following standard protocols. Reverse transcription of miRNA was carried out using a miRNA-specific reverse transcription kit, while mRNA was reverse-transcribed using a cDNA synthesis kit. Quantitative PCR was performed using SYBR Green chemistry on a real-time PCR system. U6 snRNA and GAPDH were used as internal controls for miRNA and mRNA analyzes, respectively. Relative expression levels were calculated using the 2^-^ΔΔCt method. ELK1 forward, 5′-GCTGCCTCCTAGCATTCACTTC-3′; ELK1 reverse, 5′-CCACGCTGATAGAAGGGATGTG-3′. miR-2110 forward, 5′-TGCGGTTGGGGAAACGGCCGCTG-3′; miR-2110 reverse, 5′-CCAGTGCAGGGTCCGAGGT-3′; U6 forward, 5′-GCTCGCTTCGGCAGCACA-3′; U6 reverse, 5′-AACGCTTCACGAATTTGCGTG-3′. U6 was used as the internal reference for miRNA quantification. GAPDH forward, 5′-GTCTCCTCTGACTTCAACAGCG-3′; GAPDH reverse, 5′-ACCACCCTGTTGCTGTAGCCAA-3′.

### Dual-luciferase reporter assay

Wild-type and mutant fragments of the ELK1 3′ untranslated region (3′UTR) containing the predicted miR-2110 binding sites were cloned into a luciferase reporter vector. AML cells were co-transfected with reporter constructs and miR-2110 mimics or negative controls. Luciferase activity was measured 48 h after transfection using a dual-luciferase reporter assay system, and firefly luciferase activity was normalized to Renilla luciferase activity.

### Cell viability assay

Cell viability was assessed using the Cell Counting Kit-8 (CCK-8). Transfected cells were seeded into 96-well plates and incubated for the indicated time points. CCK-8 reagent was added, and absorbance was measured at 450 nm. For drug sensitivity assays, cells were treated with increasing concentrations of Ara-C, and dose–response curves were generated.

### Establishment of Ara-C-resistant cell lines

Ara-C–resistant THP-1 and HL-60 cell lines were established by continuous exposure to gradually increasing concentrations of Ara-C over several weeks. Resistance was confirmed by CCK-8 assays comparing drug sensitivity between parental and resistant cells.

### Western blot analysis

Cells were lysed using RIPA buffer supplemented with protease and phosphatase inhibitors. Protein concentrations were quantified using a BCA assay. Equal amounts of protein were separated by SDS–PAGE and transferred to PVDF membranes. Membranes were blocked with 5% skim milk and incubated overnight at 4 °C with primary antibodies against ELK1, Cyclin D1, CDK4, P21, Bcl-2, Bax, cleaved caspase-3, Caspase-3, PARP cleaved PARP, phosphorylated ERK1, ERK1, c-FOS, EGR1, γH2AX, p-p53, and β-actin. After incubation with appropriate secondary antibodies, signals were detected using enhanced chemiluminescence. Band intensities were quantified using ImageJ software.

### Drug sensitivity and rescue experiments

To evaluate the contribution of ELK1 to miR-2110–mediated effects, AML cells were transfected with miR-2110 mimics alone or in combination with ELK1 overexpression plasmids, followed by Ara-C treatment. Cell viability and protein expression changes were assessed to determine partial rescue effects.

### Xenograft tumor model

All animal experiments were approved by the Animal Ethics Committee of Guizhou Medical University (approval number: 2402684). Female BALB/c nude mice (4–6 weeks old) were obtained from an accredited laboratory animal center and maintained under specific pathogen-free conditions with controlled temperature (22 ± 2 °C), a 12 h light/dark cycle, and free access to food and water. Nude mice were selected due to their immunodeficient background (lack of functional T cells), which allows efficient engraftment and growth of human leukemia cells *in vivo*. Although acute myeloid leukemia is a disseminated hematological malignancy, subcutaneous xenograft models are widely used as a simplified and reproducible system for evaluating tumor growth and treatment response in leukemia-related studies, particularly in mechanistic investigations (19, 20). THP-1 cells transfected with negative control or miR-2110 mimics were collected during the logarithmic growth phase, washed, and resuspended in serum-free medium. To facilitate tumor formation from suspension leukemia cells, a total of 5 × 10^6^ cells in 100 μL of a PBS/Matrigel mixture (1:1, v/v) were subcutaneously injected into the flank of each mouse to establish xenograft tumors (n = 5 per group). Tumor growth and body weight were measured every 3–4 days. Tumor volume was calculated using the formula: V = (length × width²)/2. Mice were monitored daily for general condition and tumor burden. For tumor implantation, mice were anesthetized using isoflurane inhalation anesthesia (2–3% for induction and 1–2% for maintenance in oxygen). Matrigel was included to provide an extracellular matrix-like scaffold that supports the survival, retention, and local proliferation of suspension leukemia cells following subcutaneous injection, thereby facilitating tumor establishment *in vivo*. At the experimental endpoint, mice were euthanized by carbon dioxide inhalation (gradual fill method) followed by cervical dislocation to ensure death. Tumor growth data are presented as mean ± SD, and statistical comparisons between groups were performed using two-tailed Student’s t-test. Each mouse was considered an independent biological replicate.

### Humane endpoints and animal welfare

Humane endpoints were established to minimize animal suffering during the xenograft experiments. The duration of the animal experiment was approximately 15 days after tumor cell implantation. Mice were monitored daily for tumor size, body weight, and overall health status, including activity, posture, grooming behavior, and signs of distress. Animals were euthanized if any of the following criteria were met: tumor diameter exceeding 1.5 cm, body weight loss greater than 20%, impaired mobility, persistent lethargy, ulceration at the tumor site, or other signs of severe distress. Once animals reached the predefined humane endpoint criteria, euthanasia was performed within 24 hours. A total of 10 mice were used in this study (5 per group). At the experimental endpoint, all mice were euthanized for tumor collection. No animals were found dead prior to reaching the humane endpoint criteria during the course of the study. For tumor implantation procedures, mice were anesthetized using isoflurane inhalation anesthesia (2–3% for induction and 1–2% for maintenance in oxygen). At the experimental endpoint, mice were euthanized by carbon dioxide inhalation (gradual fill method, 20–30% chamber volume per minute) followed by cervical dislocation to ensure death. Animals were maintained under specific pathogen-free (SPF) conditions, with controlled temperature and a 12 h light/dark cycle and free access to food and water. All efforts were made to minimize suffering and distress throughout the study. All animal procedures were performed by trained laboratory personnel experienced in animal handling and welfare, following institutional guidelines approved by the Animal Ethics Committee of Guizhou Medical University (approval number: 2402684).

### Statistical analysis

All experiments were performed independently at least three times. Data are presented as mean ± standard deviation (SD). Statistical analyzes were conducted using GraphPad Prism software (version 9.0; GraphPad Software, San Diego, CA, USA). Comparisons between two groups were performed using a two-tailed Student’s t-test. For multiple group comparisons, one-way analysis of variance (ANOVA) followed by Tukey’s *post hoc* test was applied. A P value < 0.05 was considered statistically significant.

## Results

### miR-2110 is aberrantly upregulated in acute myeloid leukemia

**Figure 1:**
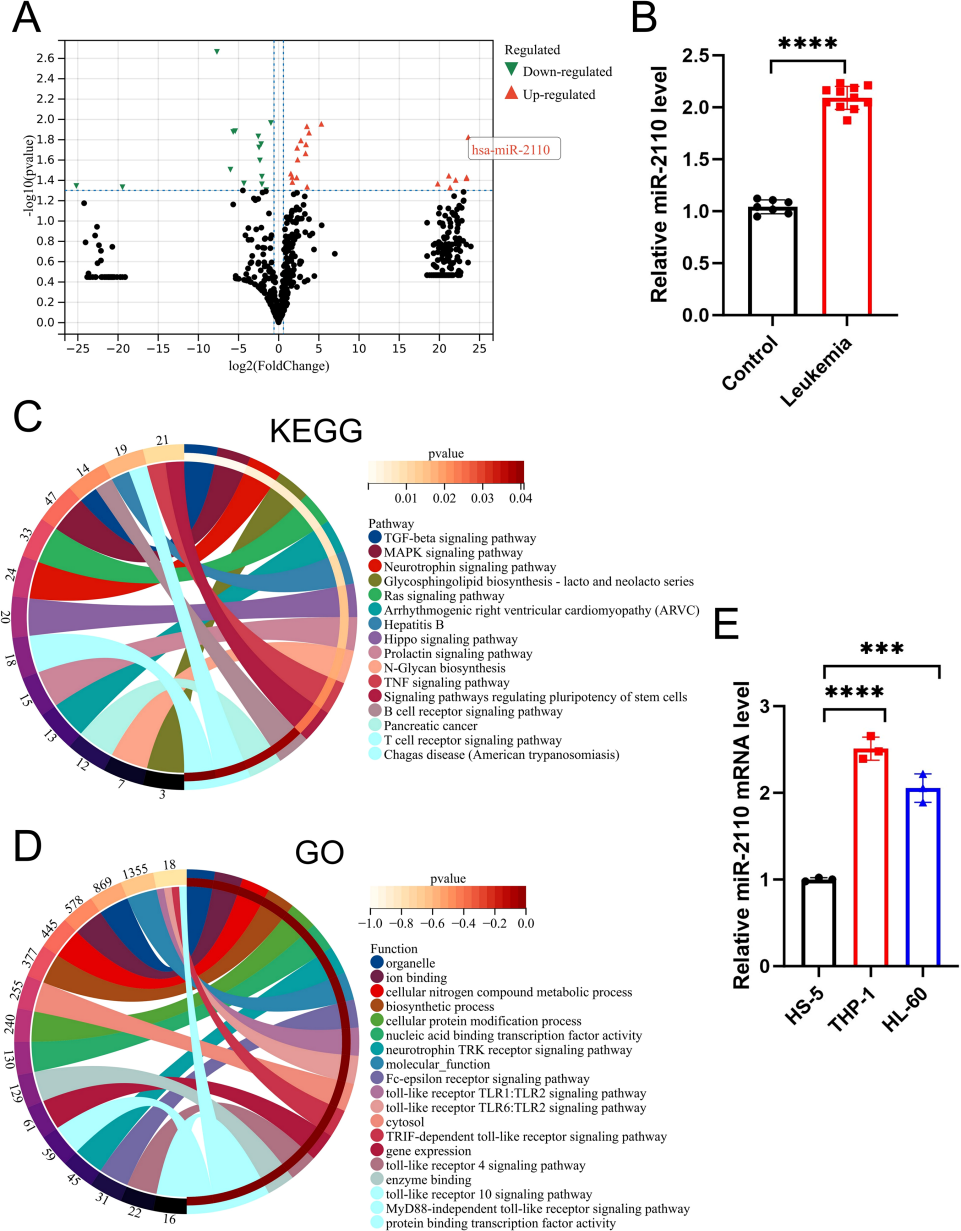
Upregulation of miR-2110 in pediatric acute myeloid leukemia and functional enrichment analysis. **(A)** Volcano plot depicting differentially expressed miRNAs between pediatric AML samples and umbilical cord blood controls from the GSE196886 dataset. miR-2110 is highlighted among significantly upregulated miRNAs. **(B)** Relative expression level of miR-2110 in pediatric AML samples compared with cord blood controls. **(C)** KEGG pathway enrichment analysis of predicted miR-2110 target genes, showing enrichment in cancer-related and signal transduction pathways. **(D)** Gene ontology enrichment analysis of miR-2110 target genes, highlighting biological processes related to transcriptional regulation and metabolic signaling. **(E)** qRT-PCR validation of miR-2110 expression in AML cell lines (THP-1, HL-60) and normal bone marrow stromal cells (HS-5). Data are presented as mean ± SD. For panel B, the control group was used as the reference and set to 1.0; control, n = 6; leukemia, n = 8. For panel E, HS-5 cells were used as the reference sample and set to 1.0; qRT-PCR experiments were independently repeated three times. Statistical significance was determined using two-tailed Student’s t-test for two-group comparisons or one-way ANOVA followed by Tukey’s *post hoc* test for multiple-group comparisons. ***P < 0.001; ****P < 0.0001.

To explore aberrantly expressed microRNAs in acute myeloid leukemia, miRNA sequencing data were obtained from the GSE196886 dataset, which comprises AML samples and umbilical cord blood controls. Differential expression analysis revealed a distinct miRNA expression landscape between leukemia and control groups, among which miR-2110 was identified as one of the most prominently upregulated miRNAs in pediatric AML samples (**Figure 1A**). Notably, several miRNAs exhibited extreme log2 fold change values, which are likely attributable to very low or near-zero expression levels in one of the comparison groups, a phenomenon commonly observed in small RNA sequencing analyzes.

Quantitative comparison further confirmed that miR-2110 expression was significantly elevated in leukemia samples relative to cord blood controls, indicating a leukemia-associated upregulation pattern (**Figure 1B**). To gain insight into the potential biological implications of miR-2110 dysregulation, predicted target genes were subjected to pathway enrichment analyzes. KEGG analysis demonstrated that miR-2110–related targets were enriched in multiple oncogenic and signal transduction pathways, including MAPK, TGF-β, Ras, Hippo, and TNF signaling pathways, all of which have been implicated in leukemogenesis (**Figure 1C**).

Consistently, Gene Ontology enrichment analysis indicated that these target genes were predominantly involved in transcriptional regulation, RNA metabolic processes, nucleic acid biosynthesis, protein binding, and immune-related receptor signaling, suggesting a broad regulatory role of miR-2110 in transcriptional and signaling networks (**Figure 1D**).

To further validate the expression pattern of miR-2110 at the cellular level, qRT-PCR analysis was performed in human AML cell lines. miR-2110 expression was markedly increased in THP-1 and HL-60 cells compared with normal bone marrow stromal cells (HS-5), with THP-1 cells exhibiting the highest expression level (**Figure 1E**). Collectively, these findings indicate that miR-2110 is consistently upregulated in pediatric AML and AML cell models, supporting its potential involvement in leukemia-related molecular processes.

### Over expression of miR-2110 suppresses proliferation and induces apoptosis in AML cells

**Figure 2:**
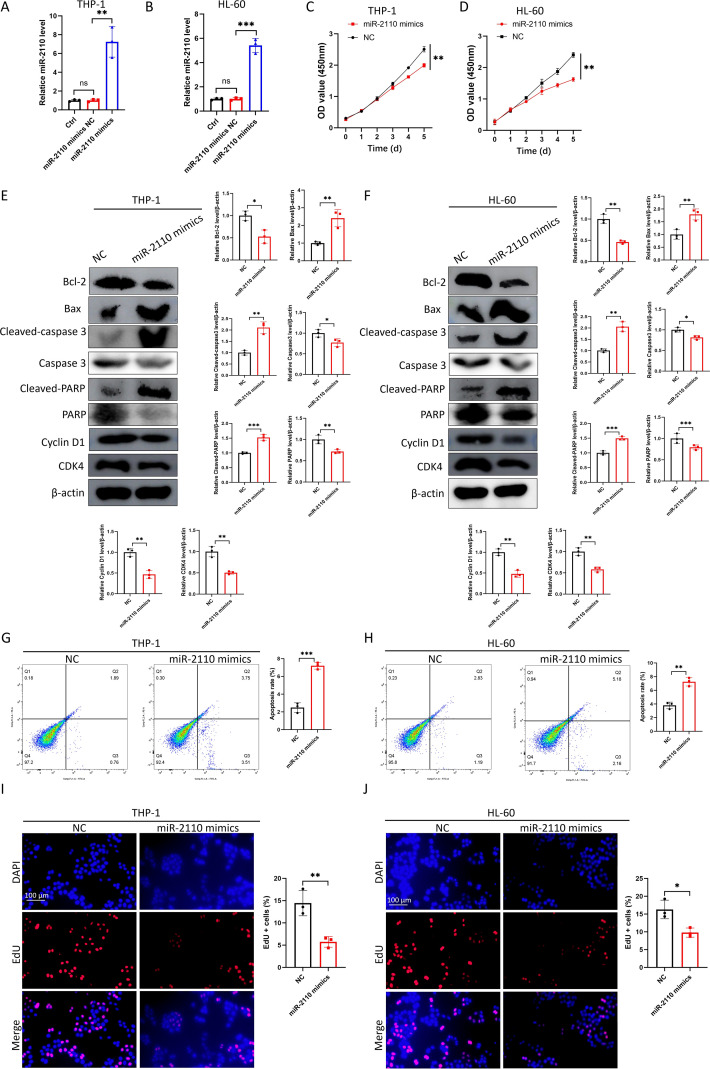
miR-2110 suppresses proliferation and induces apoptosis-related changes in AML cells. **(A, B)** qRT-PCR analysis confirming miR-2110 overexpression in THP-1 and HL-60 cells following transfection with miR-2110 mimics. The NC group was used as the reference and set to 1.0. **(C, D)** CCK-8 assays showing reduced cell viability in THP-1 and HL-60 cells upon miR-2110 overexpression over a five-day period. **(E, F)** Western blot analysis and corresponding quantification of apoptosis- and cell-cycle–related proteins in THP-1 and HL-60 cells transfected with miR-2110 mimics or negative control. β-actin was used as the loading control, and band intensities were normalized to β-actin and expressed as fold change relative to the NC group, which was set to 1.0. **(G, H)** Flow cytometry analysis of apoptosis in THP-1 **(G)** and HL-60 **(H)** cells after transfection with miR-2110 mimics or NC. The percentage of apoptotic cells is summarized in the corresponding bar charts. **(I, J)** EdU incorporation assays assessing proliferative activity in THP-1 **(I)** and HL-60 **(J)** cells. Representative images and quantitative analysis of EdU-positive cells are shown. Scale bar = 100 μm. Data are presented as mean ± SD from three independent experiments unless otherwise indicated. Statistical significance was determined using two-tailed Student’s t-test for two-group comparisons or one-way ANOVA followed by Tukey’s *post hoc* test for multiple-group comparisons. ns, not significant; *P < 0.05; **P < 0.01; ***P < 0.001.

To investigate the functional impact of miR-2110 in AML cells, miR-2110 mimics were transfected into THP-1 and HL-60 cell lines. Quantitative PCR confirmed a robust increase in miR-2110 expression following mimic transfection in both cell models, whereas no significant change was observed in negative control groups, indicating effective and specific overexpression (**Figures 2A, B**).

Cell viability was subsequently assessed using CCK-8 assays over a five-day period. Enforced expression of miR-2110 significantly attenuated proliferative capacity in both THP-1 and HL-60 cells compared with negative controls, with growth suppression becoming more pronounced over time, suggesting a sustained inhibitory effect on leukemic cell expansion (**Figures 2C, D**).

To further delineate the underlying cellular processes, apoptosis- and cell-cycle–related proteins were examined by immunoblotting. miR-2110 overexpression led to a marked reduction in the anti-apoptotic protein Bcl-2, accompanied by increased expression of pro-apoptotic Bax, cleaved caspase-3, and cleaved PARP in both THP-1 and HL-60 cells, indicating activation of the intrinsic apoptotic pathway (**Figures 2E, F**). In parallel, key regulators of G1/S cell-cycle progression, including Cyclin D1 and CDK4, were significantly downregulated following miR-2110 overexpression, consistent with cell-cycle arrest at the G1 phase (**Figures 2E, F**).

Consistent with these findings, flow cytometry analysis revealed that the proportion of apoptotic cells was significantly increased in miR-2110-overexpressing THP-1 and HL-60 cells compared with controls (**Figures 2G, H**).

Furthermore, EdU incorporation assays demonstrated that miR-2110 overexpression significantly reduced the percentage of proliferating cells in both cell lines (**Figures 2I, J**), further confirming its inhibitory effect on cell proliferation.

Collectively, these findings demonstrate that miR-2110 exerts a tumor-suppressive effect in AML cells by simultaneously inhibiting proliferation, inducing apoptosis, and disrupting cell-cycle progression.

### Integrative target prediction identifies ELK1 as a high-confidence downstream effector of miR-2110

**Figure 3:**
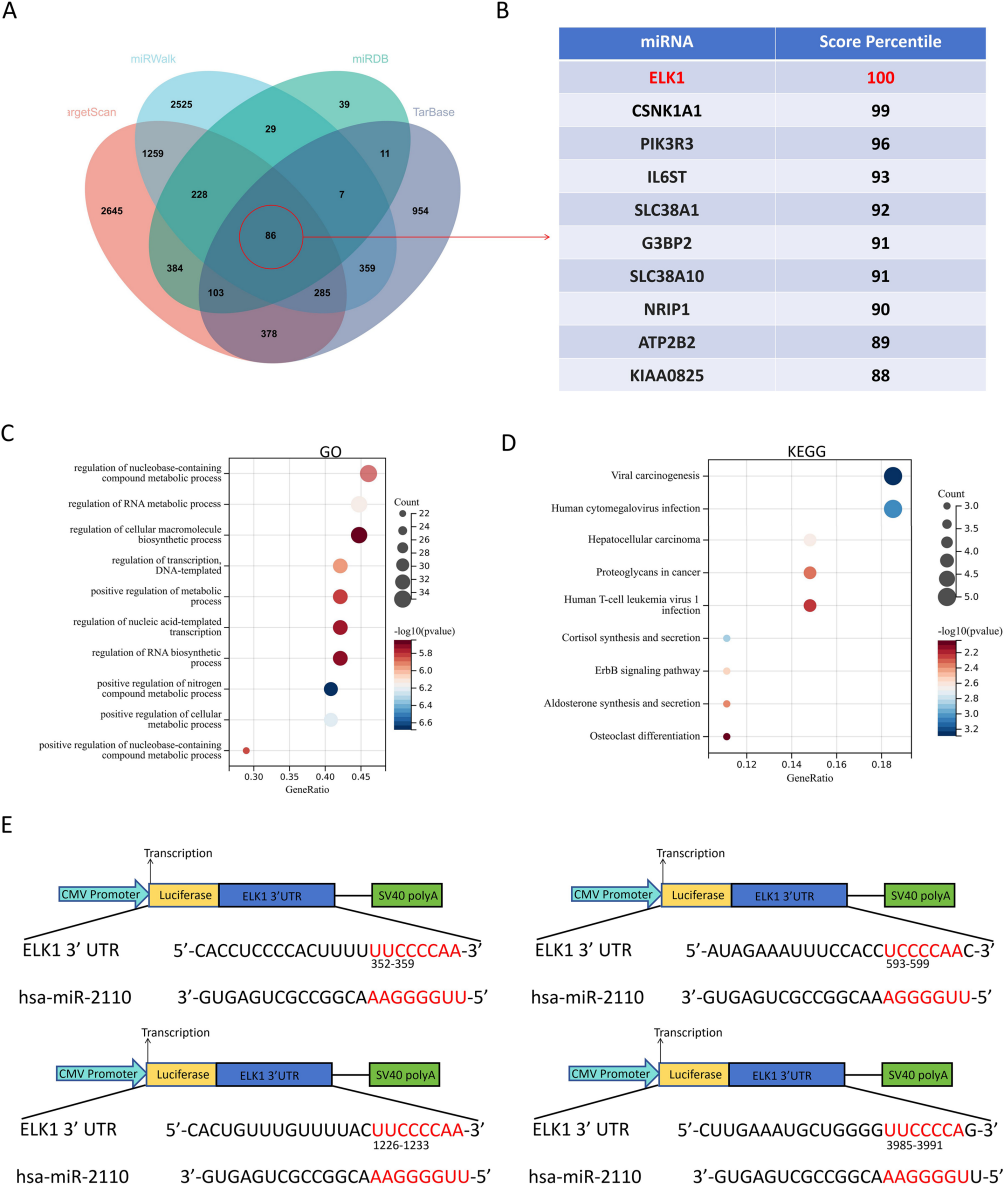
Identification of miR-2110 target genes and functional enrichment analysis. **(A)** Overlap of predicted target genes of miR-2110 from multiple databases. **(B)** Top-ranked candidate target genes based on integrated scoring. **(C–D)** GO and KEGG enrichment analyzes of predicted target genes, suggesting potential involvement in transcriptional regulation and signaling pathways. **(E)** Schematic illustration of predicted binding sites between miR-2110 and ELK1 3′UTR.

To systematically investigate the role of miR-2110 in AML, we first identified differentially expressed miRNAs from patient-derived datasets, followed by functional validation in AML cell lines, and subsequently explored downstream targets and associated signaling pathways. To identify potential downstream targets of miR-2110 in acute myeloid leukemia, a multi-database prediction strategy was employed. Candidate target genes were independently retrieved from TargetScan, miRWalk, miRDB, and TarBase, followed by intersection analysis. A total of 86 genes were consistently predicted across all four databases, representing a high-confidence candidate target set for subsequent analyzes (**Figure 3A**). To prioritize functionally relevant targets, these overlapping genes were further ranked based on integrated prediction scores derived from the respective databases, which incorporate parameters such as seed sequence complementarity, evolutionary conservation, and predicted binding affinity between miR-2110 and the 3′UTR of target mRNAs. In particular, seed region matching (positions 2–8 of the miRNA) and favorable thermodynamic stability of miRNA–mRNA duplex formation were considered key determinants of target likelihood. Based on these criteria, candidate targets were ranked using a percentile-based approach, and ELK1 exhibited the highest integrated prediction score, ranking at the 100th percentile. ELK1 also showed strong predicted binding features across multiple platforms, including conserved seed-matching sites and high context scores, compared with other candidates such as CSNK1A1, PIK3R3, and IL6ST. These results highlight ELK1 as a prioritized downstream target of miR-2110 for subsequent experimental validation (**Figure 3B**). Functional enrichment analysis was performed to explore the potential biological relevance of the predicted target genes. Gene Ontology analysis showed that these genes were primarily associated with transcriptional regulation, RNA metabolic processes, nucleobase-containing compound biosynthesis, and nucleic acid–templated transcription (**Figure 3C**). In parallel, KEGG pathway analysis suggested enrichment in cancer-related and signal transduction pathways, including viral carcinogenesis, ErbB signaling, and proteoglycans in cancer (**Figure 3D**). Based on its top-ranking prediction score and its known association with transcriptional regulation and oncogenic signaling, ELK1 was selected for further validation. To investigate the potential interaction between miR-2110 and ELK1, binding sites were predicted using the TargetScan database. To further support ELK1 as a direct target of miR-2110, putative binding sites within the ELK1 3′UTR were analyzed using TargetScan and miRWalk. A total of four candidate miR-2110 binding sites were identified across the ELK1 3′UTR. Among these, site 1 exhibited a canonical 7–8-mer seed match with favorable contextual features, including a higher TargetScan context++ score (more negative values indicate stronger predicted repression) and conservation across species, whereas the remaining sites showed comparatively weaker seed pairing or less favorable contextual scores. Consistently, miRWalk prediction further supported these interactions, with predicted minimum free energy (MFE) values indicative of stable miRNA–mRNA duplex formation. Based on these integrated criteria, site 1 was selected for subsequent validation as the most probable functional binding site (**Figure 3E**).

### miR-2110 directly targets ELK1 through a dominant functional binding site

**Figure 4:**
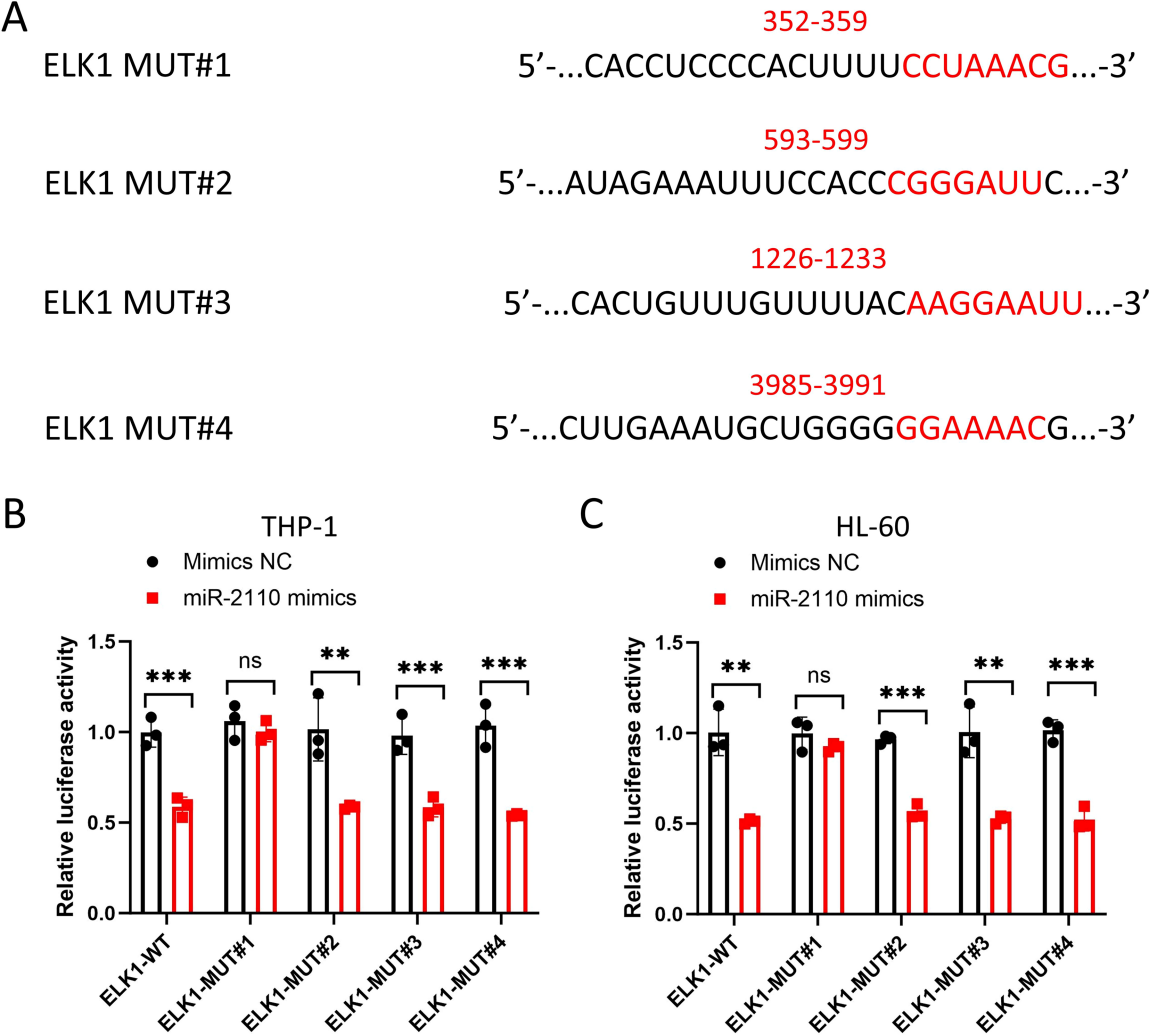
Mapping of functional miR-2110 binding sites within the ELK1 3′UTR. **(A)** Schematic illustration of mutant ELK1 3′UTR reporter constructs. Four individual mutants (MUT#1–MUT#4) were generated by introducing nucleotide substitutions into each predicted miR-2110 binding region (352–359, 593–599, 1226–1233, and 3985–3991, respectively), as indicated in red. **(B, C)** Dual-luciferase reporter assays in THP-1 **(B)** and HL-60 **(C)** cells. miR-2110 mimics significantly reduced the activity of the wild-type ELK1 3′UTR reporter. Mutation of the predicted binding sites attenuated miR-2110-mediated repression to varying degrees, with MUT#1 showing the strongest loss of responsiveness, indicating that site 1 represents the dominant functional binding site within the ELK1 3′UTR. Firefly luciferase activity was normalized to Renilla luciferase activity and expressed relative to the corresponding mimic NC group. Data are presented as mean ± SD from three independent experiments. Statistical significance was determined using two-tailed student’s t-test for comparisons between mimic NC and miR-2110 mimic groups within each reporter construct. ns, not significant; **P < 0.01; ***P < 0.001.

To dissect which predicted region within the ELK1 3′UTR is functionally responsible for miR-2110 binding, four mutant reporter constructs were generated. In each construct, only one predicted binding region was mutated while the remaining sequence was kept unchanged, including sites at positions 352–359 (MUT#1), 593–599 (MUT#2), 1226–1233 (MUT#3), and 3985–3991 (MUT#4) (**Figure 4A**).

In THP-1 cells, transfection of miR-2110 mimics significantly reduced the luciferase activity of the reporter carrying the wild-type ELK1 3′UTR. This inhibitory effect was completely abolished when the first binding site was mutated (MUT#1), whereas reporters harboring mutations at the other three sites (MUT#2, MUT#3, and MUT#4) remained responsive to miR-2110, showing a degree of repression comparable to that observed with the wild-type construct (**Figure 4B**).

A consistent pattern was observed in HL-60 cells. miR-2110 markedly suppressed the activity of the wild-type reporter, and this suppression was lost only in the MUT#1 construct, while the MUT#2, MUT#3, and MUT#4 reporters still exhibited significant decreases in luciferase activity upon miR-2110 overexpression (**Figure 4C**).

This observation suggests that site 1 represents the dominant functional binding site within the ELK1 3′UTR. The stronger regulatory effect of site 1 may be attributed to its more optimal seed sequence complementarity, favorable local sequence context, and potentially greater accessibility within the 3′UTR structure. In contrast, the remaining sites may contribute to miR-2110–mediated regulation in a supportive or cooperative manner but appear to exert relatively weaker individual effects.

### miR-2110 suppresses ELK1 expression and associates ELK1 with a MAPK-centered signaling network

**Figure 5:**
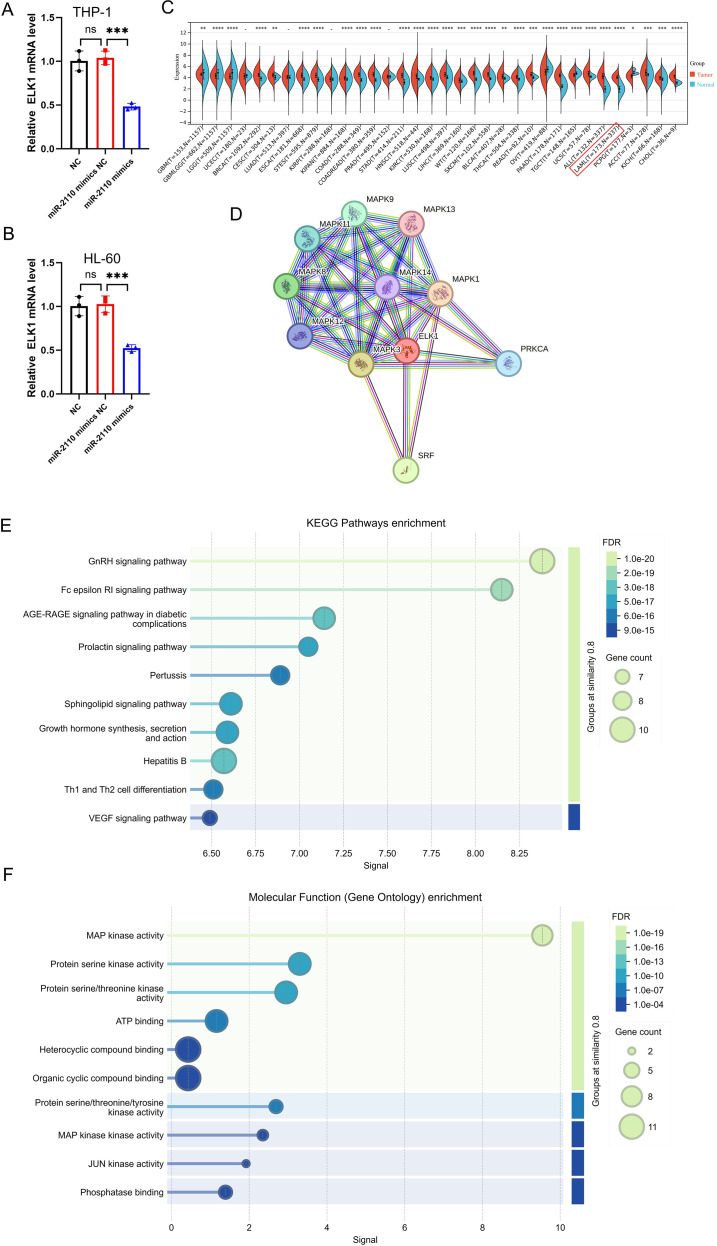
miR-2110 downregulates ELK1 and places ELK1 within a MAPK-associated interaction context. **(A, B)** qRT-PCR analysis showing reduced ELK1 mRNA levels in THP-1 and HL-60 cells after transfection with miR-2110 mimics. The NC group was used as the reference and set to 1.0. **(C)** Expression profile of ELK1 across multiple tumor and normal cohorts, showing relatively higher ELK1 expression in malignant tissues. **(D)** Protein–protein interaction network generated by querying the STRING database using ELK1 as the seed protein to identify known and predicted ELK1-associated interacting proteins in humans. **(E)** KEGG pathway enrichment analysis of ELK1-associated interacting proteins. **(F)** Gene Ontology molecular function enrichment analysis of ELK1-associated interacting proteins. Data in **(A)** and **(B)** are presented as mean ± SD from three independent experiments. Statistical significance was determined using two-tailed student’s t-test. ns, not significant; ***P < 0.001.

miR-2110 suppresses ELK1 expression and places ELK1 within a MAPK-associated signaling context. Overexpression of miR-2110 significantly reduced ELK1 mRNA levels in both THP-1 and HL-60 cells, whereas the negative control showed no detectable effect, suggesting that miR-2110 negatively regulates endogenous ELK1 expression in AML cells (**Figures 5A, B**). To further explore the potential clinical relevance of ELK1, its expression pattern was examined across multiple tumor and normal cohorts. ELK1 exhibited relatively higher expression in malignant tissues compared with corresponding normal samples, including leukemia-related datasets, indicating a potential association between ELK1 expression and tumor-related contexts (**Figure 5C**). To provide additional functional context, protein–protein interaction analysis was performed by querying the STRING database using ELK1 as the seed protein. This analysis revealed that ELK1 is connected with multiple signaling-related proteins, including members of the MAPK family, forming an interaction network (**Figure 5D**). Functional enrichment analysis of ELK1-associated interacting proteins suggested enrichment in pathways related to signal transduction and cancer-associated processes, including MAPK-related pathways (**Figure 5E**). Gene Ontology analysis further indicated enrichment in molecular functions such as kinase activity, ATP binding, and protein serine/threonine kinase activity (**Figure 5F**). Taken together, these findings suggest that miR-2110 suppresses ELK1 expression and that ELK1 is connected to signaling networks involving MAPK-related components, providing a potential functional context for the observed cellular effects, although a direct mechanistic relationship in AML requires further investigation.

### ELK1 mediates miR-2110–associated changes in cell-cycle and apoptosis-related proteins in AML cells

**Figure 6:**
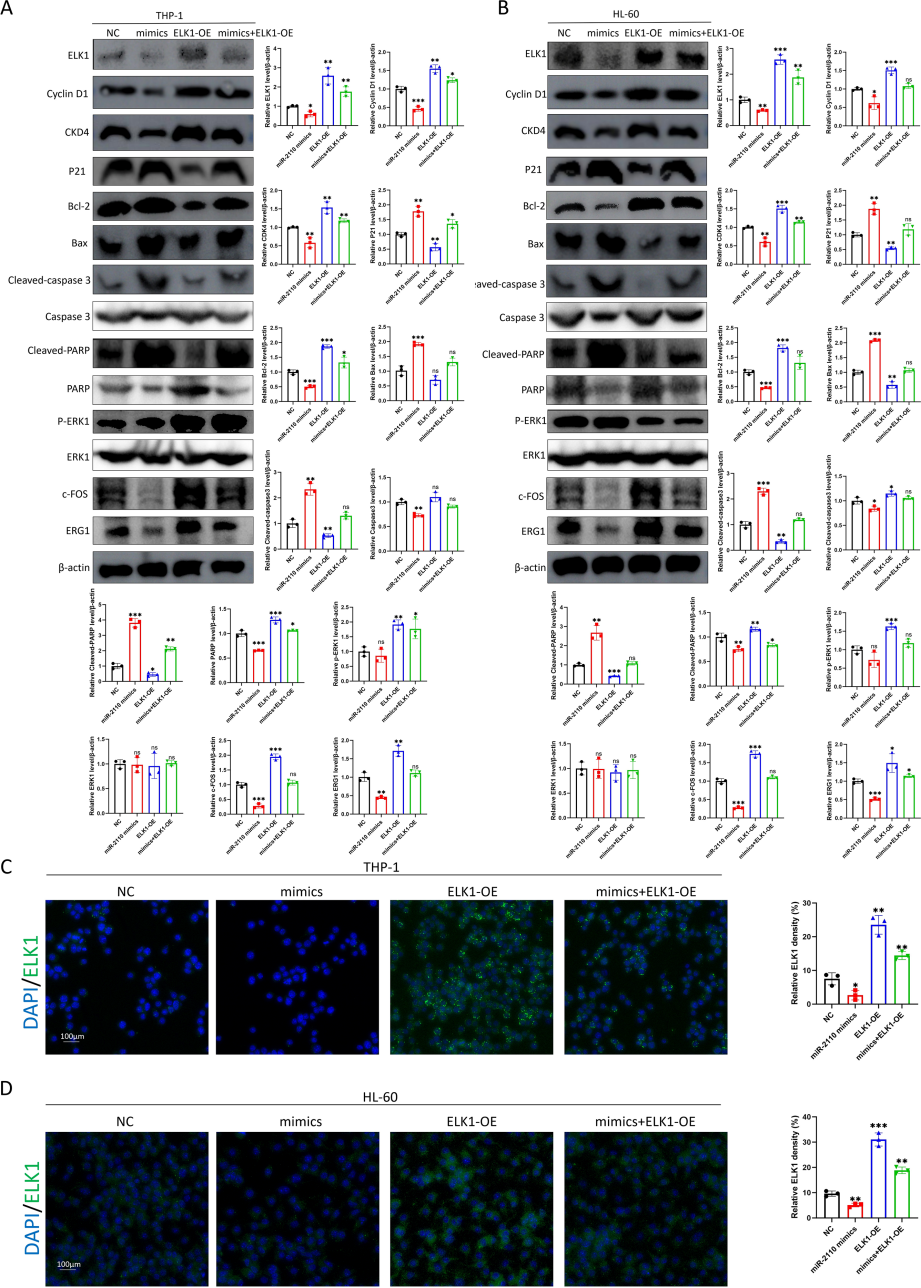
ELK1 contributes to miR-2110–associated changes in cell-cycle-, apoptosis-, and MAPK-related protein expression in AML cells. **(A)** Western blot analysis and densitometric quantification of ELK1, cell-cycle–related proteins (Cyclin D1, CDK4, and p21), apoptosis-related proteins (Bcl-2, Bax, cleaved caspase-3, total caspase-3, cleaved PARP, and total PARP), and selected MAPK-associated markers (p-ERK1, total ERK1, c-FOS, and EGR1) in THP-1 cells transfected with negative control, miR-2110 mimics, ELK1 overexpression plasmid, or miR-2110 mimics combined with ELK1 overexpression. **(B)** Corresponding western blot analysis and quantification in HL-60 cells under the same experimental conditions. β-actin was used as the loading control. For cleaved proteins and phosphorylated proteins, band intensities were normalized to the corresponding total protein levels where applicable and then expressed as fold change relative to the NC group, which was set to 1.0. Other protein levels were normalized to β-actin and expressed relative to the NC group. **(C, D)** Immunofluorescence staining of ELK1 in THP-1 and HL-60 cells following the indicated treatments. Representative images and quantitative fluorescence intensity are shown. Scale bar = 100 μm. Data are presented as mean ± SD from three independent experiments. Statistical significance was determined using one-way ANOVA followed by Tukey’s *post hoc* test. ns, not significant; *P < 0.05; **P < 0.01; ***P < 0.001.

ELK1 contributes to miR-2110–associated changes in protein regulators of cell-cycle and apoptosis in AML cells. Given that ELK1 was identified as a direct target of miR-2110 and placed within a MAPK-associated transcriptional context, we next examined whether ELK1 is involved in the molecular changes associated with miR-2110 in AML cells. To this end, ELK1 overexpression was introduced in the presence of miR-2110 mimics in THP-1 and HL-60 cells. At the protein level, miR-2110 overexpression resulted in reduced ELK1 expression, accompanied by decreased levels of Cyclin D1 and CDK4 and increased expression of the cell-cycle–related protein p21. Reintroduction of ELK1 partially restored the expression of these proteins, suggesting that ELK1 is associated with miR-2110–related changes in cell-cycle regulatory protein expression (**Figures 6A, B**). In parallel, miR-2110 mimics altered the expression of apoptosis-associated proteins, including downregulation of Bcl-2 and upregulation of Bax, cleaved caspase-3, and cleaved PARP. These changes were partially attenuated by ELK1 overexpression, indicating that ELK1 is involved in miR-2110–associated modulation of apoptosis-related protein expression (**Figures 6A, B**). Given that ELK1 functions as an ERK-responsive transcription factor within MAPK signaling, we further examined pathway-associated readouts. miR-2110 overexpression was accompanied by reduced p-ERK1 levels and decreased expression of ELK1-associated immediate-early genes, including c-FOS and EGR1. Re-expression of ELK1 partially attenuated these changes. Considering that ERK is positioned upstream of ELK1 in canonical MAPK signaling, the observed reduction in p-ERK1 should be interpreted as a pathway-associated change rather than evidence of a direct upstream regulatory effect mediated by miR-2110, and may reflect broader alterations in signaling output involving complex regulatory or feedback processes. Collectively, these results suggest that miR-2110 modulates ELK1-associated molecular signatures related to cell-cycle regulation, apoptosis, and MAPK-linked transcriptional activity in AML cells (**Figures 6A, B**) (21–25). ELK1 immunofluorescence staining further supported changes in ELK1 expression at the cellular level (**Figures 6C, D**). Overall, these findings indicate that ELK1 is involved in miR-2110–associated molecular changes rather than providing direct functional evidence of altered cell-cycle progression or apoptosis. (**Figures 6C, D**). Quantitative rescue analysis showed that ELK1 re-expression restored approximately 77.2–85.7% of the miR-2110-induced increase in cleaved caspase-3, while selected miR-2110-suppressed markers, including Cyclin D1, Bcl-2, and ELK1-associated signaling readouts, were restored to or slightly above baseline levels. These findings indicate that ELK1 makes a substantial contribution to miR-2110-associated molecular changes, although incomplete rescue of apoptosis-related markers suggests that additional miR-2110 targets may also be involved. Overall, these findings indicate that ELK1 is involved in miR-2110–associated molecular changes rather than providing direct functional evidence of altered cell-cycle progression or apoptosis.

### miR-2110 enhances Ara-C responsiveness and partially counteracts Ara-C resistance in AML cells

**Figure 7:**
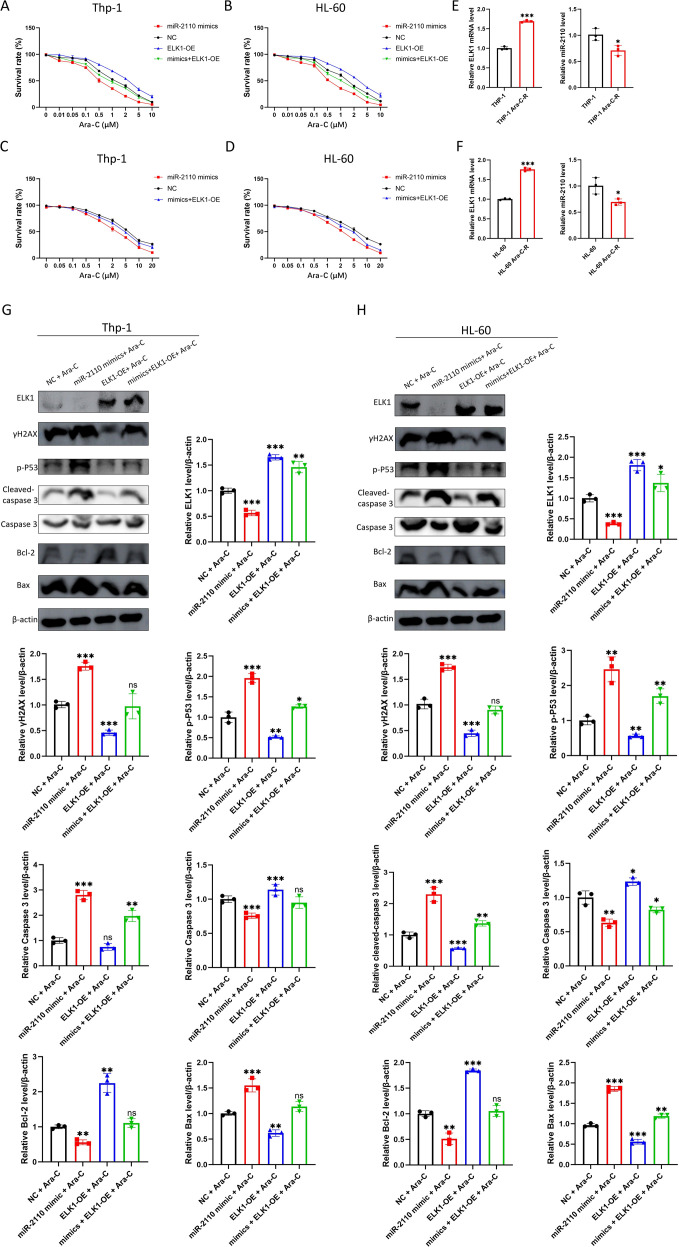
miR-2110 enhances Ara-C responsiveness and partially counteracts Ara-C resistance in AML cells. **(A, B)** Ara-C dose–response curves showing survival rates of parental THP-1 and HL-60 cells transfected with negative control, miR-2110 mimics, ELK1 overexpression plasmid, or miR-2110 mimics combined with ELK1 overexpression. **(C, D)** Ara-C dose–response curves in Ara-C–resistant THP-1 and HL-60 cells under the indicated transfection conditions. Cell survival was measured using CCK-8 assays and expressed relative to untreated cells in each corresponding group. **(E, F)** qRT-PCR analysis of endogenous ELK1 mRNA and miR-2110 expression in parental and Ara-C-resistant THP-1 cells **(E)** and HL-60 cells **(F)**. THP-1/Ara-C-R and HL-60/Ara-C-R indicate Ara-C-resistant THP-1 and HL-60 cells, respectively. Parental cells were used as the reference and set to 1.0. GAPDH was used as the internal control for ELK1 mRNA quantification, and U6 was used as the internal control for miR-2110 quantification. **(G, H)** Western blot analysis and densitometric quantification of ELK1, γH2AX, total H2AX, phosphorylated p53, total p53, cleaved caspase-3, total caspase-3, Bcl-2, and Bax in THP-1 and HL-60 cells treated with Ara-C. β-actin was used as the loading control. γH2AX and phosphorylated p53 were normalized to total H2AX and total p53, respectively; cleaved caspase-3 was normalized to total caspase-3; other proteins were normalized to β-actin. Values were expressed as fold change relative to the corresponding NC group, which was set to 1.0. Data are presented as mean ± SD from three independent experiments. Statistical significance was determined using one-way ANOVA followed by Tukey’s *post hoc* test for multi-group comparisons. For dose–response curves, statistical comparisons were performed at the indicated concentrations. ns, not significant; *P < 0.05; **P < 0.01; ***P < 0.001.

Given that miR-2110–ELK1 signaling influenced proliferative and apoptotic features in AML cells, we next examined whether this axis was also associated with cellular responsiveness to Ara-C. Dose–response analyzes were first performed in parental THP-1 and HL-60 cells. Overexpression of miR-2110 produced a leftward shift of the Ara-C survival curves, indicating increased drug sensitivity, whereas ELK1 overexpression showed an opposite tendency. Reintroduction of ELK1 partially weakened the sensitizing effect of miR-2110, supporting a contributory role of ELK1 in the regulation of Ara-C response (**Figures 7A, B**).

To further assess the relevance of miR-2110 under conditions of acquired drug resistance, Ara-C–resistant THP-1 and HL-60 cell models were examined. In both resistant cell lines, miR-2110 overexpression partially restored responsiveness to Ara-C, as reflected by a shift of the dose–response curves toward lower concentrations. This effect was attenuated by ELK1 re-expression, suggesting that ELK1 participates in the maintenance of reduced Ara-C responsiveness in resistant AML cells (**Figures 7C, D**).

To further characterize the Ara-C-resistant AML cell models, endogenous miR-2110 and ELK1 mRNA levels were examined in parental and Ara-C-resistant THP-1 and HL-60 cells. Compared with parental THP-1 cells, THP-1/Ara-C-R cells showed significantly increased ELK1 mRNA expression and reduced miR-2110 expression (**Figure 7E**). A similar expression pattern was observed in HL-60/Ara-C-R cells, with elevated ELK1 mRNA and decreased miR-2110 levels compared with parental HL-60 cells (**Figure 7F**). These findings suggest that downregulation of miR-2110 and upregulation of ELK1 are associated with the Ara-C-resistant phenotype, further supporting the relevance of the miR-2110–ELK1 axis in Ara-C responsiveness.

At the molecular level, Ara-C–treated AML cells were analyzed for selected markers related to DNA damage and apoptosis. miR-2110 overexpression was associated with increased γH2AX accumulation, enhanced p53 phosphorylation, elevated cleaved caspase-3, and reduced Bcl-2 expression after Ara-C exposure. In contrast, ELK1 overexpression mitigated these changes, whereas co-expression of miR-2110 and ELK1 produced intermediate effects (**Figures 7G, H**). Although Ara-C is generally considered an S-phase–active drug, these findings suggest that, in the present model, the enhanced drug sensitivity observed after miR-2110 overexpression may be more closely related to increased susceptibility to DNA damage and apoptosis than to proliferative status alone (26, 27).

Collectively, these findings indicate that miR-2110 is associated with enhanced Ara-C responsiveness in both parental and resistant AML cells, and that ELK1 contributes, at least in part, to this phenotype.

### miR-2110 restrains tumor progression *in vivo*

**Figure 8:**
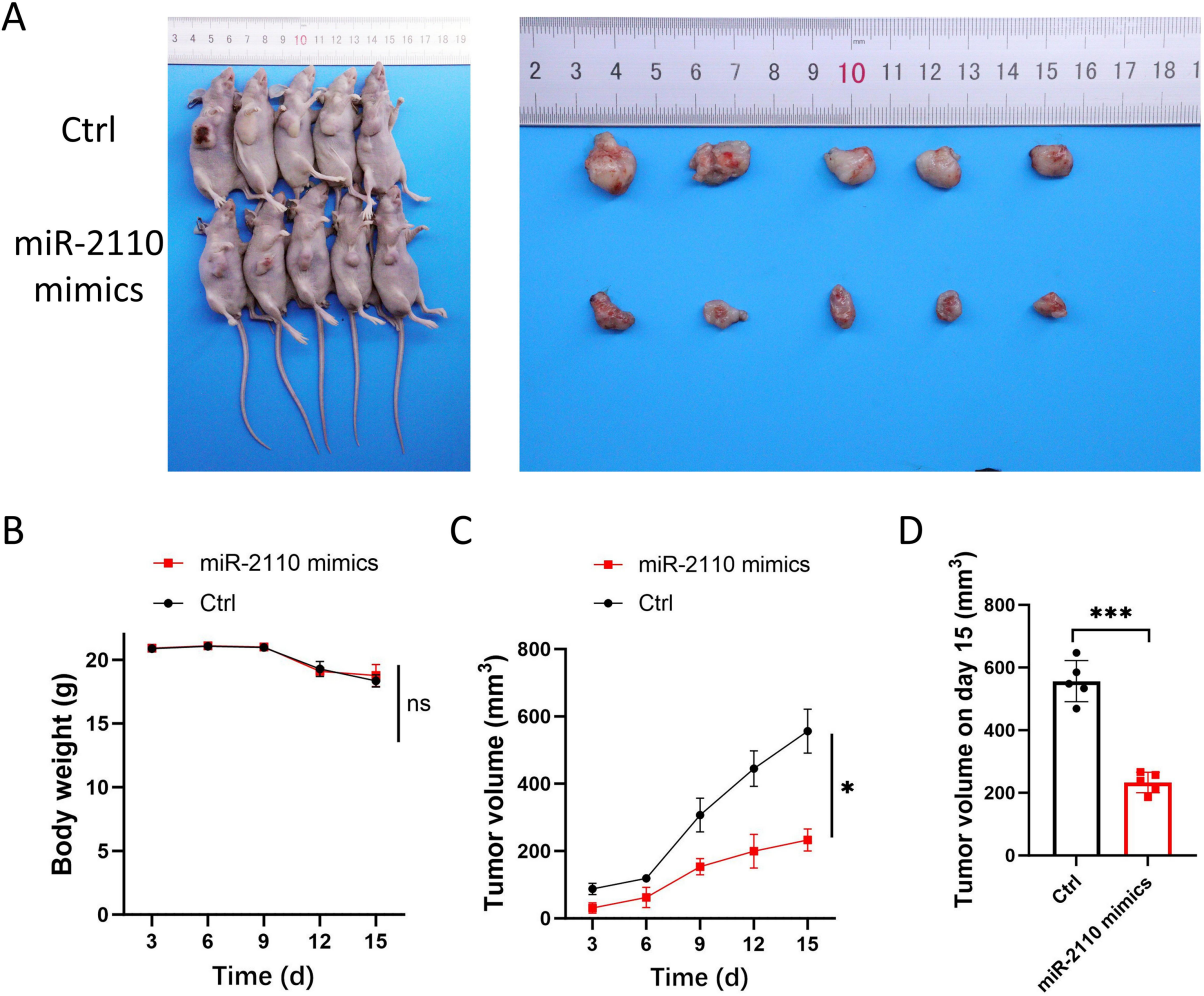
miR-2110 suppresses xenograft tumor growth without obvious body weight loss. **(A)** Representative images of nude mice bearing subcutaneous tumors and isolated tumors from the NC and miR-2110 mimics groups. THP-1 cells transfected with NC or miR-2110 mimics were subcutaneously injected into BALB/c nude mice to establish xenograft tumors. **(B)** Body weight monitoring during tumor growth. No significant difference in body weight was observed between the two groups. **(C)** Tumor growth curves showing tumor volumes measured at the indicated time points. **(D)** Comparison of tumor volumes at the experimental endpoint between the NC and miR-2110 mimics groups. Data are presented as mean ± SD (n = 5 mice per group). Tumor volume was calculated as V = (length × width²)/2. Statistical significance was determined using two-tailed student’s t-test for endpoint comparisons and repeated-measures analysis or two-way ANOVA for tumor growth curves, where appropriate. ns, not significant; *P < 0.05; ***P < 0.001.

To evaluate the biological effect of miR-2110 *in vivo*, a subcutaneous xenograft model was established using AML cells carrying either the negative control or miR-2110 mimics. Gross observation showed that tumors formed in the miR-2110 mimics group were visibly smaller than those in the control group, and excised tumor specimens further confirmed a marked reduction in tumor mass (**Figure 8A**).

During the entire experimental period, body weight was continuously recorded to assess general health status. No obvious difference in body weight was detected between the two groups at any time point, indicating that miR-2110 treatment did not cause detectable systemic burden or overt toxicity in the host animals (**Figure 8B**).

Dynamic measurement of tumor volumes revealed a significantly slower growth rate in the miR-2110 mimics group compared with the control group. The inhibitory effect became more pronounced as the experiment progressed, demonstrating a sustained suppressive influence of miR-2110 on tumor expansion (**Figure 8C**). Consistently, tumors harvested at day 15 from mice receiving miR-2110 mimics exhibited substantially smaller volumes than those from the control group (**Figure 8D**).

Together, these findings demonstrate that miR-2110 exerts a strong tumor-suppressive effect *in vivo* while maintaining a favorable safety profile, providing supportive evidence for its antileukemic potential.

### Graphical summary of the miR-2110–ELK1 signaling axis in AML

**Figure 9:**
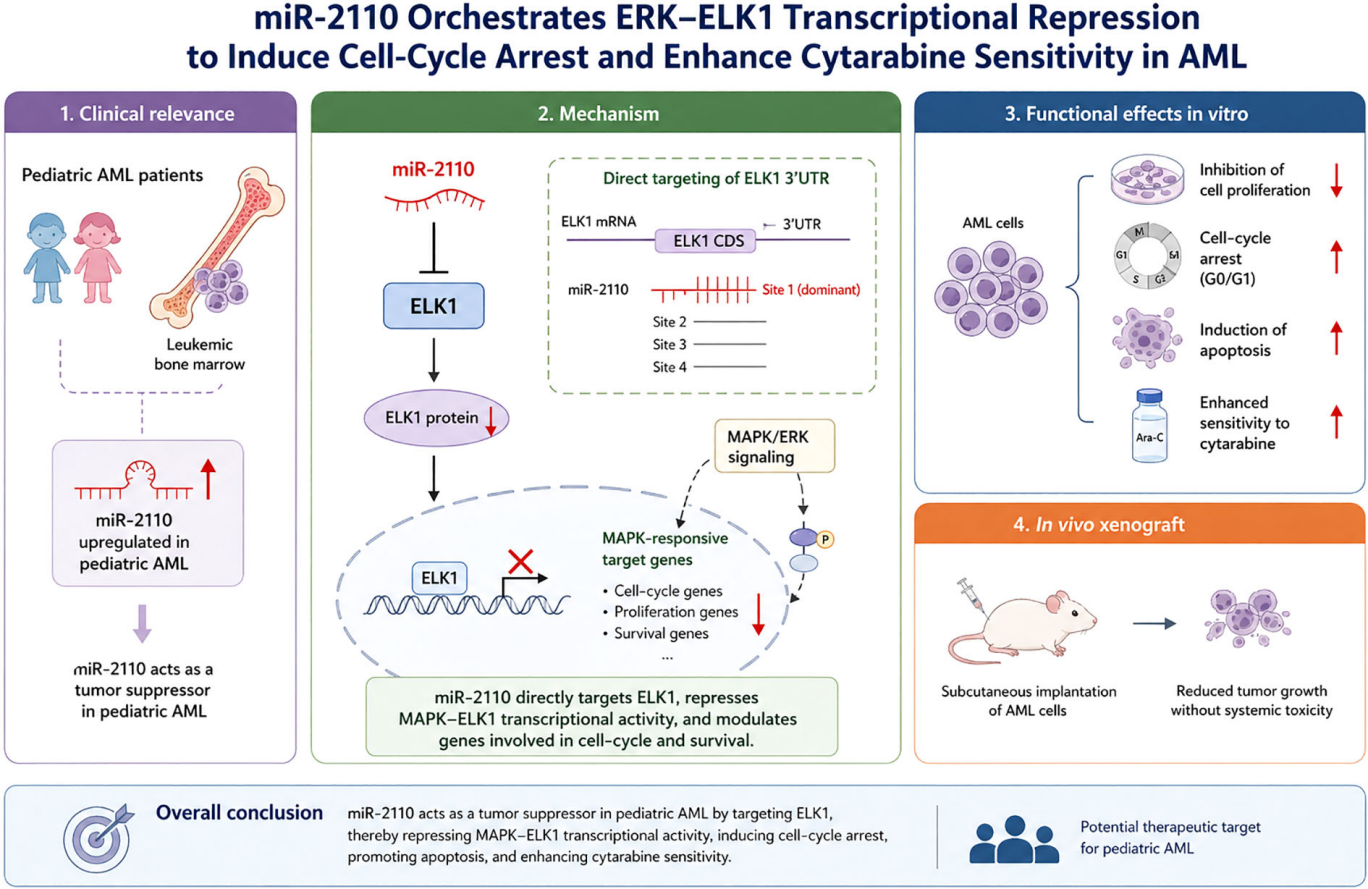
Graphical abstract illustrating the miR-2110–ELK1 regulatory axis in acute myeloid leukemia. miR-2110 is upregulated in pediatric AML and functions as a tumor suppressor by directly targeting the 3′UTR of ELK1, leading to reduced ELK1 expression and suppression of MAPK/ERK-associated transcriptional activity. This regulation results in inhibition of cell proliferation, induction of cell-cycle arrest, promotion of apoptosis, and increased sensitivity to Ara-C in AML cells. *In vivo*, miR-2110 overexpression suppresses tumor growth in a xenograft model. Overall, miR-2110–ELK1 signaling represents a potential therapeutic target in AML.

To provide an integrated overview of the study findings, a graphical abstract summarizing the miR-2110–ELK1 regulatory axis was constructed (**Figure 9**). As illustrated, miR-2110 directly targets ELK1 and attenuates MAPK-associated transcriptional activity, leading to reduced proliferation, cell-cycle arrest, enhanced apoptosis, and increased Ara-C sensitivity in AML cells. These observations are further supported by *in vivo* data demonstrating suppressed tumor growth. Collectively, these findings highlight the functional significance of miR-2110 in AML and its potential therapeutic relevance.

## Discussion

In this study, we systematically investigated the role of miR-2110 in acute myeloid leukemia and found that miR-2110 exerted tumor-suppressive effects despite being upregulated in pediatric AML. By integrating patient-derived miRNA sequencing data with functional assays, target validation, network-based analyzes, and drug response evaluation, we identified a miR-2110–ELK1 regulatory axis associated with leukemic cell survival and Ara-C responsiveness.

MicroRNAs are widely recognized as key post-transcriptional regulators in AML, with established roles in controlling proliferation, apoptosis, and therapeutic response. Dysregulation of miRNA networks has been implicated in leukemogenesis as well as treatment resistance, highlighting their dual roles as functional regulators and potential therapeutic targets. One notable finding is that miR-2110 is elevated in pediatric AML samples while functionally restraining leukemic cell growth (28–30). This apparent paradox highlights an important feature of miRNA biology, namely that increased miRNA abundance does not necessarily equate to oncogenic function. In malignant settings, some miRNAs may be induced as part of adaptive or stress-responsive programs that partially counterbalance excessive oncogenic signaling (31, 32). Our data suggest that miR-2110 upregulation in AML may represent an insufficient compensatory mechanism that fails to fully counterbalance oncogenic signaling inputs. Several non-mutually exclusive explanations may account for this paradoxical observation. First, miR-2110 upregulation may represent a compensatory response induced by oncogenic stress, inflammatory pressure, or treatment-related cellular stress, rather than a primary oncogenic driver. Second, increased miR-2110 expression at the bulk-sample level may reflect subclonal heterogeneity within pediatric AML, in which different leukemic cell populations display distinct stress-adaptive regulatory states. Third, in drug-resistant or highly proliferative leukemic cells, feedback activation of survival pathways may override the inhibitory effects of tumor-suppressive miRNAs, resulting in a state in which miR-2110 is elevated but functionally insufficient to fully restrain leukemic progression. This interpretation is consistent with the broader concept that miRNA expression in cancer is context-dependent and may participate in complex regulatory networks rather than simply acting as either oncogenic or tumor-suppressive in a linear manner (31, 33).

Through integrative bioinformatic prediction and experimental validation, ELK1 was confirmed as a direct downstream target of miR-2110. ELK1 is a well-characterized MAPK-responsive effector protein involved in mediating downstream cellular responses to extracellular stimuli (34, 35). Our network-based analyzes further positioned ELK1 at the center of multiple MAPK-associated signaling components, supporting the possibility that miR-2110 modulates AML cell behavior, at least in part, through attenuation of MAPK-related signaling output. Importantly, rescue experiments showed that enforced ELK1 expression only partially reversed the effects of miR-2110 on cell-cycle regulators, apoptotic markers, and MAPK-associated proteins, indicating that ELK1 is an important mediator of miR-2110 function but is unlikely to be its sole downstream effector. The quantitative rescue analysis further supports this interpretation. ELK1 re-expression restored several miR-2110-suppressed markers to or slightly above baseline levels, whereas the miR-2110-induced increase in cleaved caspase-3 was only partially reversed. This pattern suggests that ELK1 makes a substantial contribution to the miR-2110-associated molecular phenotype, but does not fully account for all downstream effects. Such incomplete rescue is biologically plausible because individual miRNAs usually regulate multiple target transcripts and may produce phenotypic outcomes through coordinated modulation of a broader regulatory network. In line with this possibility, enrichment analyzes implicated additional cancer-related pathways, including Ras-, TGF-β-, Hippo-, TNF-, and MAPK-associated signaling. These pathways should be interpreted as hypothesis-generating rather than experimentally confirmed mechanisms in the present study. Future systematic gain- and loss-of-function screening will be needed to determine whether additional miR-2110 targets cooperate with ELK1 in shaping AML cell proliferation, apoptosis, and Ara-C responsiveness.

Importantly, miR-2110 enhanced the responsiveness of AML cells to Ara-C in both parental and drug-resistant models. Ara-C resistance remains a major clinical challenge in AML, and its underlying determinants are complex and multifactorial (36–38). Our findings indicate that miR-2110 increases Ara-C-induced growth inhibition and cell death, accompanied by enhanced DNA damage signaling and apoptotic activation. Reintroduction of ELK1 partially mitigated these effects, supporting the involvement of the miR-2110–ELK1 axis in modulating drug sensitivity rather than acting as a standalone determinant of resistance.

An additional point that merits consideration is the apparently paradoxical relationship between reduced proliferative activity and increased Ara-C sensitivity. Ara-C is classically regarded as an S-phase–active chemotherapeutic agent, and rapidly cycling cells are generally considered more susceptible to its cytotoxic effects. However, our data suggest that, in the setting of miR-2110 overexpression, the overall response to Ara-C cannot be explained solely by proliferative status. Although miR-2110 reduced EdU incorporation and downregulated Cyclin D1/CDK4, it simultaneously enhanced Ara-C–associated γH2AX accumulation, p53 activation, and apoptotic signaling (26, 27). These observations support the interpretation that the net increase in drug sensitivity may reflect a greater vulnerability to DNA damage and apoptosis, which outweighs the reduction in proliferative drive. Therefore, in this context, miR-2110 appears to shift AML cells toward a state that is less proliferative but more permissive to Ara-C–induced cytotoxic stress.

Although AML is a disseminated hematologic malignancy, subcutaneous xenograft models using human AML cell lines have been used in previous mechanistic and therapeutic studies as practical systems for monitoring tumor growth and treatment response. For example, AML xenograft studies have established subcutaneous tumors using HL-60 or MV4–11 cells in immunodeficient mice, and Matrigel-assisted implantation has also been used to support leukemia cell engraftment in this setting. For suspension leukemia cells, Matrigel can provide an extracellular matrix-like scaffold that facilitates local cell retention and tumor establishment after implantation. Nevertheless, this model cannot capture key features of systemic AML biology, including bone marrow engraftment, peripheral blood dissemination, leukemic burden, marrow microenvironmental interactions, and survival-related outcomes. Therefore, the *in vivo* data in the present study should be interpreted as supportive evidence of tumor growth suppression rather than definitive evidence of anti-leukemic activity in a systemic AML setting. Future studies using systemic or orthotopic leukemia models, such as NSG- or NSGS-based engraftment systems, will be needed to determine whether miR-2110 affects leukemic dissemination, bone marrow infiltration, and overall survival (19, 39).

miR-2110 has been reported in several solid tumor contexts, including neuroblastoma and nasopharyngeal carcinoma, where it has been associated with tumor-suppressive biological effects (40, 41). However, its role in AML remains poorly characterized. Therefore, the novelty of the present study lies in identifying miR-2110 as an upregulated but functionally tumor-suppressive miRNA in AML, and in linking it to ELK1 regulation, apoptotic signaling, and Ara-C responsiveness. This provides a disease-specific context for miR-2110 that has not been well explored previously.

The GSE196886 dataset was selected because it specifically contains miRNA sequencing data from pediatric AML samples and umbilical cord blood controls, which matches the focus of the present study. Compared with broader leukemia datasets or datasets lacking suitable controls, GSE196886 provides a direct framework for identifying pediatric AML-associated miRNA alterations. Nevertheless, we acknowledge that the dataset lacks detailed matched clinical and molecular annotations, and future studies using larger well-annotated cohorts will be necessary to validate the clinical significance of miR-2110.

Ara-C was selected because it remains a backbone chemotherapeutic agent in AML treatment. As a deoxycytidine analog, Ara-C is converted intracellularly to its active triphosphate form and incorporated into DNA during replication, thereby inhibiting DNA synthesis and promoting DNA damage-associated cell death. Since treatment failure and relapse in AML are closely related to Ara-C resistance, evaluating whether miR-2110 affects Ara-C responsiveness provides clinically relevant information regarding its potential role in therapeutic sensitivity (42).

The present study mainly used miR-2110 mimics and ELK1 overexpression as gain-of-function approaches. Although these approaches are useful for evaluating the biological consequences of increased miR-2110 or ELK1 activity, they do not fully reflect endogenous regulatory dynamics. Loss-of-function strategies, including miR-2110 inhibitors, LNA-mediated silencing, CRISPR-based gene perturbation, or stable lentiviral overexpression/knockdown systems, would further strengthen causal interpretation. Future studies incorporating these approaches will be important to validate the endogenous role of miR-2110 and ELK1 in AML.

ELK1 was prioritized not only because it was predicted and experimentally validated as a miR-2110 target, but also because it is functionally linked to MAPK-associated signaling networks (43, 44). STRING-based interaction analysis placed ELK1 in proximity to several MAPK-related components, including MAPK1, MAPK3, MAPK8, MAPK11, MAPK13, MAPK14, SRF, and PRKCA. These proteins are involved in signaling processes that connect extracellular stimuli to gene expression programs, including immediate early gene regulation (45). However, these findings should be interpreted as providing functional context rather than proving a direct AML-specific MAPK mechanism. In addition to MAPK-associated signaling, enrichment analyzes also suggested potential involvement of other cancer-related pathways, including Ras, TGF-β, Hippo, and TNF signaling. These pathways are closely related to cell survival, apoptotic regulation, inflammatory responses, and cellular adaptation in malignant contexts. However, these results should be interpreted as hypothesis-generating rather than definitive mechanistic evidence. Given the multi-target nature of miRNA regulation, it is plausible that miR-2110 may influence AML cell behavior through a broader regulatory network beyond ELK1 alone. In the present study, ELK1 was prioritized as a representative high-confidence target because of its strong prediction score, multiple validated 3′UTR binding sites, and biological relevance to the observed phenotypes. Future studies using systematic screening or parallel validation of additional candidate targets will be needed to determine whether Ras-, TGF-β-, Hippo-, or TNF-related pathways cooperate with ELK1 in mediating the effects of miR-2110.

Several limitations of this study should be acknowledged. Although ELK1 was validated as a direct target of miR-2110, additional downstream mediators are likely involved in shaping the observed phenotypes. Moreover, while MAPK-associated signaling changes were documented, we did not attempt to delineate upstream regulatory hierarchies or global downstream programs regulated by ELK1. These aspects warrant further investigation but were beyond the scope of the current work. Moreover, currently available public datasets lack comprehensive annotations linking miRNA expression with functional phenotypes such as cell proliferation, apoptosis, MAPK signaling activity, and treatment response. Therefore, future studies using well-characterized clinical cohorts will be necessary to further evaluate the clinical significance of miR-2110 in AML. Besides, we acknowledge that the subcutaneous xenograft model does not fully reflect the disseminated characteristics of AML. Future studies using systemic leukemia models, such as NSG- or NSGS-based engraftment systems, will be important to further evaluate the effects of miR-2110 on leukemic progression and survival. In addition, the current study mainly used miR-2110 mimics as a gain-of-function approach. Although this strategy is useful for evaluating the biological consequences of increased miR-2110 levels, it does not fully reflect endogenous miRNA regulation. Future studies using miR-2110 inhibitors, LNA-based silencing, or stable lentiviral systems would further clarify the endogenous role of miR-2110 in AML.

In addition to miR-2110, the differential expression analysis identified several other dysregulated miRNAs in the dataset, including both upregulated and downregulated candidates. However, the present study focused on miR-2110 based on its consistent expression pattern, relatively high fold change, and the limited prior characterization in AML, which provided an opportunity for mechanistic exploration. We acknowledge that other differentially expressed miRNAs may also contribute to leukemogenesis or therapeutic response. Importantly, these additional miRNAs were not incorporated as experimental controls in the current study, as the primary objective was to characterize the functional role of a single candidate miRNA in depth rather than perform comparative analyzes across multiple miRNAs. Future studies incorporating systematic screening or parallel functional validation of multiple candidates would be valuable to further delineate the broader miRNA regulatory landscape in AML.

In conclusion, our study identifies miR-2110 as a tumor-suppressive miRNA in pediatric AML that modulates leukemic cell survival and chemotherapeutic response through partial repression of ELK1 and MAPK-associated signaling effects. By combining patient-derived data with functional and drug-sensitivity analyzes, this work moves beyond descriptive miRNA profiling and provides mechanistic insight into how miRNA-mediated regulation can influence leukemia biology and treatment response.

## Data Availability

The original contributions presented in the study are included in the article/supplementary material. Further inquiries can be directed to the corresponding author.

## Glossary

AML: Acute myeloid leukemia
Ara-C: Cytarabine
BCA: Bicinchoninic acid
CCK-8: Cell Counting Kit-8
CDK4: Cyclin-dependent kinase 4
c-FOS: FBJ murine osteosarcoma viral oncogene homolog
EGR1: Early growth response 1
ELK1: ETS-like transcription factor 1
ERK: Extracellular signal-regulated kinase
GEO: Gene Expression Omnibus
HL-60: Human promyelocytic leukemia cell line
KEGG: Kyoto Encyclopedia of Genes and Genomes
MAPK: Mitogen-activated protein kinase
miRNA: MicroRNA
NC: Negative control
PARP: Poly(ADP-ribose) polymerase
PBS: Phosphate-buffered saline
PCR: Polymerase chain reaction
PVDF: Polyvinylidene fluoride
RIPA: Radioimmunoprecipitation assay
SDS-PAGE: Sodium dodecyl sulfate–polyacrylamide gel electrophoresis
THP-1: Human monocytic leukemia cell line
WB: Western blot
